# Exploratory benchmarking of AI-generated diet plans for inherited protein metabolism disorders: a simulation-based evaluation of nutritional accuracy and clinical safety

**DOI:** 10.3389/fnut.2026.1877144

**Published:** 2026-08-26

**Authors:** Taha Gökmen Ülger, Emre Adıgüzel

**Affiliations:** 1 Department of Nutrition and Dietetics, Bolu Abant Izzet Baysal University, Bolu, Türkiye; 2 Department of Nutrition and Dietetics, Karamanoğlu Mehmetbey University, Karaman, Türkiye

**Keywords:** artificial intelligence, inherited metabolic disorders, maple syrup urine disease, medical nutrition therapy, phenylketonuria, propionic acidemia

## Abstract

**Objective:**

Artificial intelligence (AI)-based large language models (LLMs) are increasingly used to support nutrition-related decision-making; however, their ability to generate clinically appropriate dietary plans for inherited protein metabolism disorders remains largely unexplored. This study aimed to perform an exploratory simulation-based benchmarking analysis of AI-generated dietary plans for phenylketonuria (PKU), maple syrup urine disease (MSUD), and propionic acidemia (PPA) using disease-specific metabolic nutrition guidelines.

**Methods:**

Standardized pediatric case scenarios were developed for PKU, MSUD, and PPA. Using identical English-language prompts, ChatGPT-5.3 Pro and Gemini 3 Pro Advanced each generated 3-day dietary plans. Nutrient composition was analyzed using the BeBiS Nutrition Information System and evaluated against Dietary Reference Intakes (DRIs). Disease-specific nutritional targets, including amino acid intake, protein distribution, and energy provision, were benchmarked against recommendations from Genetic Metabolic Dietitians International (GMDI). Nutritional characteristics of the dietary plans generated by the two AI models were compared using exploratory statistical analyses.

**Results:**

Both LLMs generated structured dietary plans with generally acceptable overall nutritional characteristics; however, clinically relevant deviations from disease-specific nutritional targets were identified across all three disorders. In the PKU case, both models achieved the recommended phenylalanine range, but neither simultaneously met protein and tyrosine recommendations. In the MSUD case, differences were primarily related to energy provision and branched-chain amino acid targets, while in the PPA case neither model achieved the recommended balance between intact protein and total protein. These findings demonstrated that conventional measures of nutritional adequacy alone were insufficient to determine the clinical appropriateness of AI-generated dietary plans for inherited protein metabolism disorders.

**Conclusion:**

General-purpose LLMs can generate structured dietary plans for inherited protein metabolism disorders; however, disease-specific metabolic targets are not consistently achieved. Evaluation of AI-generated dietary plans should therefore extend beyond conventional nutritional assessment and incorporate disease-specific benchmarking against established metabolic nutrition guidelines. This study provides a disease-specific benchmarking framework for evaluating AI-generated dietary plans in inherited protein metabolism disorders.

## Introduction

Inherited metabolic disorders (IMDs) involving protein and amino acid metabolism are among the most complex conditions in clinical nutrition because dietary treatment requires not only adequate energy and nutrient intake, but also precise regulation of disease-specific amino acid targets, medical formula use, and ongoing biochemical monitoring (1, 2). In disorders such as phenylketonuria (PKU), maple syrup urine disease (MSUD), and propionic acidemia (PPA), nutritional management directly influences metabolic stability, growth, neurodevelopment, and the prevention of acute or chronic complications (3–5). Therefore, dietary planning in these conditions differs fundamentally from general pediatric diet planning, as apparently adequate energy or total protein intake may still be metabolically unsafe if critical amino acid targets are not achieved.

The PKU, MSUD, and PPA were selected for the present study because they represent three clinically distinct but nutritionally high-risk models of inherited protein metabolism disorders. In PKU, dietary treatment requires restriction of phenylalanine while ensuring adequate protein-equivalent intake, tyrosine provision, and use of phenylalanine-free medical foods or low-protein products to support growth and neurodevelopment (6, 7). In MSUD, the primary dietary challenge is the careful regulation of branched-chain amino acids, particularly leucine, isoleucine, and valine, together with sufficient energy intake to prevent endogenous protein catabolism and metabolic decompensation (8, 9). In PPA, nutritional therapy requires a balance between restricting propiogenic amino acid load and maintaining adequate intact protein, total protein, and energy intake to support anabolism and growth (10, 11). These examples illustrate why disease-specific metabolic safety cannot be inferred from general dietary reference standards alone.

In recent years, artificial intelligence (AI)-based systems and large language models (LLMs) have increasingly been explored in nutrition and dietetics, including dietary assessment, menu planning, precision nutrition, patient education, and decision-support applications (12–14). These technologies may offer practical advantages by rapidly generating individualized dietary suggestions and integrating patient-specific information into nutrition-related outputs (15, 16). However, the use of general-purpose LLMs in specialized clinical nutrition raises important safety and reproducibility concerns. Unlike rule-based clinical decision-support systems, LLMs may generate fluent and clinically plausible responses without necessarily ensuring biochemical accuracy, guideline adherence, or reproducibility across repeated prompts and model versions (17, 18).

This distinction is particularly important in inherited metabolic disorders, where the clinical consequences of dietary error may be more severe than in general nutrition counseling. For example, a diet plan may appear nutritionally adequate in terms of total energy, total protein, or micronutrient intake, yet remain unsafe if it provides insufficient tyrosine in PKU, inadequate isoleucine or valine balance in MSUD, or excessive total protein relative to propiogenic amino acid tolerance in PPA (5, 19). Thus, evaluating AI-generated diet plans in this context requires a framework that distinguishes general nutritional adequacy from disease-specific metabolic safety.

Previous studies have demonstrated the potential of artificial intelligence to support dietary assessment, nutrition education, personalized nutrition, and dietary planning in a variety of clinical and non-clinical settings (20–23). However, these investigations have largely focused on general dietary recommendations, chronic disease management, or overall diet quality. To the best of our knowledge, no previous study has systematically evaluated the nutritional accuracy and clinical safety of large language model (LLM)-generated diet plans for inherited protein metabolism disorders by benchmarking their outputs against disease-specific metabolic nutrition guidelines. Consequently, it remains unclear whether general-purpose LLMs can generate dietary plans that satisfy the highly specific nutritional and metabolic requirements of disorders such as PKU, MSUD, and PPA, where precise control of amino acid intake, medical formula use, and metabolic balance is essential.

Therefore, the present study was designed as an exploratory, simulation-based benchmarking analysis to evaluate the nutritional accuracy and clinical safety of AI-generated diet plans for inherited protein metabolism disorders. Using standardized pediatric case scenarios representing PKU, MSUD, and PPA, diet plans generated by two widely used general-purpose LLMs were systematically compared with disease-specific metabolic nutrition recommendations and dietary reference standards. Rather than establishing definitive superiority of one model over another, this study aimed to identify clinically meaningful deviations from guideline-based nutritional targets, evaluate the potential metabolic safety implications of these deviations, and provide an initial benchmarking framework for the assessment of AI-generated dietary recommendations in rare inherited metabolic disorders.

## Materials and methods

### Study design

This study was designed as an exploratory, simulation-based benchmarking analysis to evaluate the nutritional accuracy and potential clinical safety of AI-generated dietary plans for inherited protein metabolism disorders. Rather than determining the superiority or intrinsic performance of individual large language models (LLMs), the study aimed to assess the extent to which AI-generated dietary recommendations complied with disease-specific metabolic nutrition guidelines and general dietary reference standards under standardized conditions.

The methodological framework consisted of three sequential stages: (i) development of standardized pediatric clinical case scenarios, (ii) generation of dietary plans using two general-purpose LLMs under identical prompting conditions, and (iii) evaluation of the generated dietary plans through quantitative nutrient analysis and disease-specific clinical benchmarking. Accordingly, the unit of evaluation was the AI-generated dietary plan, while nutritional adequacy was assessed using Dietary Reference Intakes (DRIs) and disorder-specific recommendations published by Genetic Metabolic Dietitians International (GMDI).

Because the primary objective was to benchmark AI-generated dietary recommendations rather than to evaluate within-model variability, one standardized pediatric case scenario was developed for each disorder, and each AI model generated one complete 3-day dietary plan for each case. All models were evaluated using identical clinical information, standardized prompts, and the same output requirements to ensure methodological consistency across comparisons. Therefore, the findings should be interpreted within the context of this exploratory simulation and the specific model versions evaluated rather than as definitive evidence of generalizable model behavior.

For this study, ChatGPT-5.3 Pro (OpenAI) and Gemini 3 Pro Advanced (Google) were selected because they are among the most widely used publicly accessible general-purpose LLMs. Dietary plans were generated using the web-based interfaces of ChatGPT Pro and Gemini Advanced on 16 February 2026. Default model settings were used, and no model parameters were manually modified. Each prompt was entered into a new independent conversation to eliminate the influence of previous chat history. No follow-up prompts, output regeneration, or manual optimization requests were performed after the initial response. The first complete dietary plan generated by each model was retained for nutritional analysis.

### Establishing case scenarios and prompt design

Synthetic pediatric case scenarios were developed for each disorder based on the available literature and current disease-specific nutritional management guidelines. Phenylketonuria (PKU), maple syrup urine disease (MSUD), and propionic acidemia (PPA) were selected because they represent clinically distinct inherited protein metabolism disorders requiring different dietary management strategies and precise disease-specific nutritional interventions. Each case scenario included standardized clinical information, including diagnosis, age, sex, height, body weight, and disorder-specific biochemical status. The use of synthetic cases enabled standardized benchmarking of AI-generated dietary plans while avoiding ethical concerns associated with the use of real patient data (16).

To minimize variability arising from age, sex, and growth status, all case scenarios were standardized to represent a 5-year-old boy with anthropometric measurements corresponding to the 50th percentile of the World Health Organization Child Growth Standards (height: 110 cm; body weight: 18.3 kg) (24). This standardized reference case was selected to provide a clinically representative pediatric scenario while ensuring methodological consistency across all evaluated disorders and AI models.

Dietary plans generated by ChatGPT 5.3 and Gemini 3 Pro were developed using identical standardized clinical scenarios and identical prompts. The prompts were written in English using clinically oriented language to minimize ambiguity and to ensure that both models received exactly the same clinical information and instructions (16, 25). Each prompt was entered into a new independent conversation, thereby eliminating the influence of previous conversation history. No follow-up prompts, clarification requests, output regeneration, or manual modifications were performed after the initial response. Consequently, the first complete dietary plan generated by each model was retained for analysis.

The primary prompt requested a complete 3-day dietary plan, including all meals, food items, and portion sizes or weights. To reduce unnecessary variability between outputs, both models were instructed to present their responses using the same predefined output format. Furthermore, the prompt specified that all disorder-specific biochemical parameters were within normal clinical limits. These included phenylalanine and tyrosine concentrations for PKU (26), branched-chain amino acids, ketone bodies, and ammonia concentrations for MSUD (27), and bicarbonate, lactate, and ammonia concentrations for PPA (28).

A second predefined prompt was used only when a medical formula was included in the dietary plan. This prompt requested the commercial product name and the prescribed amount of the medical formula to enable accurate nutrient analysis. No additional prompts were used to modify or optimize the dietary plans generated by either model. The complete prompts used for each disorder are presented in Table 1.

**Table 1:** Standardized prompts used in the exploratory simulation-based benchmarking of AI-generated 3-day dietary plans for PKU, MSUD, and PPA.

Metabolic condition	Prompts	
PKU	Prompt-1	Can you plan a 3-day sample menu for a 5-year-old male child with phenylketonuria, whose height is 110 cm and body weight is 18.3 kg, taking into account his energy and nutrient requirements? The levels of all blood biochemical parameters, including phenylalanine and tyrosine, are within normal limits. Please specify the portions and grammages of the ingredients for all meals.
	Prompt-2	Could you specify the brand of the medical formula and the dosage for each meal?
MSUD	Prompt-1	Can you plan a 3-day sample menu for a 5-year-old male child with maple syrup urine disease, whose height is 110 cm and body weight is 18.3 kg, taking into account his energy and nutrient requirements? The levels of all blood biochemical parameters, including branched-chain amino acids, ketone bodies, and ammonia, are within normal limits. Please specify the portions and grammages of the ingredients for all meals.
	Prompt-2	Could you specify the brand of the medical formula and the dosage for each meal?
PPA	Prompt-1	Can you plan a 3-day sample menu for a 5-year-old male child with propionic acidemia, whose height is 110 cm and body weight is 18.3 kg, taking into account his energy and nutrient requirements? The levels of all blood biochemical parameters, including bicarbonate, lactate, and ammonia, are within normal limits. Please specify the portions and grammages of the ingredients for all meals.
	Prompt-2	Could you specify the brand of the medical formula and the dosage for each meal?

### Analysis of dietary plans

After the AI-generated dietary plans were obtained (Appendix 1), their energy and nutrient contents were independently analyzed by two researchers experienced in clinical nutrition using the BeBiS (Nutrition Information System) software (29). Any discrepancies regarding food selection, portion size, or product coding were resolved through consensus before the final analysis. BeBiS was selected because it is a widely used nutritional analysis software in clinical nutrition research and contains a comprehensive food composition database adapted for the Turkish population.

When AI-generated food items did not exactly correspond to entries in the BeBiS database, the closest nutritionally equivalent food item was selected according to standard clinical dietetic practice. Portion sizes reported as household measures were converted to gram weights using standardized conversion tables when necessary. Composite dishes were coded according to their constituent ingredients whenever direct database entries were unavailable. Medical formulas were entered into BeBiS using the commercial products specified by the AI models and their prescribed amounts. When low-protein specialty foods recommended by the AI models were unavailable in the BeBiS database, nutrient values were entered using manufacturer-provided nutritional information or, where appropriate, the closest nutritionally equivalent product.

All nutrient values were compared with the age- and sex-specific Dietary Reference Intakes (DRIs) established by the National Institutes of Health (30), and the results were expressed as the percentage of the DRI (DRI%). The DRI comparison was used to evaluate overall nutritional adequacy, whereas disease-specific metabolic requirements were assessed separately using guideline-based clinical benchmarking, as described below. Accordingly, the DRI analysis was not intended to determine metabolic suitability for inherited protein metabolism disorders but rather to provide a standardized reference for evaluating general nutrient adequacy.

Because amino acid composition may vary according to the availability of food composition data, amino acid findings were interpreted in conjunction with disease-specific clinical recommendations rather than in isolation.

### Disease-specific clinical benchmarking

Because general Dietary Reference Intakes (DRIs) do not account for the disease-specific metabolic requirements of inherited protein metabolism disorders, additional disease-specific benchmarking was performed using recommendations published by Genetic Metabolic Dietitians International (GMDI), an internationally recognized organization specializing in metabolic nutrition therapy (31). The GMDI recommendations served as the primary clinical reference standards for evaluating the metabolic appropriateness of AI-generated dietary plans.

For children with PKU older than 4 years, the recommended dietary phenylalanine and tyrosine intakes are 200–1,100 mg/day and 4,000–6,000 mg/day, respectively. In addition, dietary protein intake is recommended to provide 120–140% of the age-specific DRI to support normal growth and development. These parameters were used to evaluate the adequacy of phenylalanine restriction, tyrosine provision, and total protein intake in the AI-generated dietary plans.

For children with MSUD older than 4 years, the recommended dietary intakes of leucine, isoleucine, and valine are 275–500 mg/day, 250–450 mg/day, and 325–500 mg/day, respectively. Furthermore, dietary protein and energy intakes are recommended to provide approximately 120 and 100% of the age-specific DRI, respectively. These values served as disease-specific benchmark targets for assessing the nutritional suitability of the AI-generated menus.

For male children aged 4–8 years with PPA, the recommended dietary energy, intact protein, and total protein intakes are 59–88 kcal/kg/day, 0.57–0.95 g/kg/day, and 0.95–1.14 g/kg/day, respectively (Table 2). Intact protein and total protein were evaluated separately because total protein may include protein equivalents provided by medical formulas, whereas intact protein represents natural dietary protein intake.

**Table 2:** Disease-specific clinical benchmarking references for energy, protein, and amino acid intake in PKU, MSUD, and PPA according to GMDI recommendations.

	PKU	MSUD	PPA
Energy (kcal/kg/day)		DRI for age	59–88
Intact protein (g/kg/day)			0.57–0.95
Total protein (g/kg/day)	120–140% DRI for age	120% DRI for age	0.95–1.14
Phenylalanine (mg/day)	200–1,100		
Tyrosine (mg/day)	4,000–6,000		
Leucine (mg/day)		275–500	
Isoleucine (mg/day)		250–450	
Valine (mg/day)		325–500	

Overall, AI-generated dietary plans were evaluated by comparing their nutrient values with both DRIs and disease-specific GMDI recommendations. General nutritional adequacy was assessed using DRIs, whereas metabolic appropriateness was assessed according to disorder-specific guideline targets. Deviations from guideline ranges were interpreted in terms of their potential nutritional and clinical significance rather than solely on the basis of statistical significance.

### Statistical analyses

Data were analyzed using GraphPad Prism statistical software (version 10.2.0; GraphPad Software Inc., San Diego, CA, United States). Nutrient values are presented as the mean ± standard deviation (SD) of the three daily menus generated within each AI-produced 3-day dietary plan. The SD reflects day-to-day variation within each generated dietary plan rather than variability across independent experimental observations. Because each AI model generated a single standardized 3-day dietary plan for each disorder, inferential statistical testing was not performed. Instead, differences between ChatGPT- and Gemini-generated dietary plans are presented descriptively using mean ± SD, absolute differences, and relative differences (%). The objective of these descriptive comparisons was not to establish superiority of one AI model over another, but to characterize the extent to which each generated dietary plan aligned with general nutritional recommendations and disease-specific metabolic guideline targets. Overall nutritional adequacy was evaluated against DRIs, whereas clinical appropriateness was assessed through disease-specific benchmarking against recommendations from GMDI. Accordingly, interpretation focused on descriptive deviations from guideline-based nutritional targets rather than statistical inference.

## Results

Three standardized pediatric case scenarios representing phenylketonuria (PKU), maple syrup urine disease (MSUD), and propionic acidemia (PPA) were evaluated separately. For each disorder, AI-generated 3-day dietary plans were first assessed for overall nutritional adequacy using Dietary Reference Intakes (DRIs) and subsequently benchmarked against disease-specific recommendations published by Genetic Metabolic Dietitians International (GMDI). The findings are presented separately for each disorder to facilitate evaluation of general nutrient composition, disease-specific nutritional targets, and amino acid profiles.

### Phenylketonuria (PKU)

The nutritional characteristics of the AI-generated 3-day dietary plans for the standardized PKU case are summarized in Table 3. ChatGPT generated a higher mean daily energy intake than Gemini (1522.8 ± 100.2 vs. 1225.9 ± 144.8 kcal/day), corresponding to an absolute difference of 296.9 kcal/day (24.2%). Protein intake was also considerably higher in the ChatGPT-generated dietary plan, providing 243.3 ± 4.3% of the age-specific DRI compared with 90.0 ± 24.2% in the Gemini-generated plan. Among the micronutrients, the largest relative differences were observed for vitamin D, calcium, iron, zinc, magnesium, phosphorus, and vitamin B12, all of which were higher in the ChatGPT-generated dietary plan. In contrast, both models generated comparable amounts of carbohydrate, fat, vitamin A, vitamin E, total folate, vitamin C, sodium, potassium, and dietary fiber. Overall, the two AI-generated dietary plans demonstrated distinct differences in their general nutritional composition, with ChatGPT generally providing higher energy, protein, and several micronutrients than Gemini.

**Table 3:** Exploratory comparison of daily energy, macronutrient, micronutrient, and DRI% values of 3-day diet plans generated by ChatGPT and Gemini for the PKU case.

	ChatGPT	Gemini	Absolute difference	Relative difference (%)
Energy (kcal/day)	1522.8 ± 100.2	1225.9 ± 144.8	296.9	24.2
Protein (g/day)	46.2 ± 0.8	17.1 ± 4.6	29.1	170.2
Protein (DRI %)	243.3 ± 4.3	90.0 ± 24.2		
Carbohydrate (g/day)	220.2 ± 26.4	181.7 ± 7.1	38.5	21.2
Carbohydrate (DRI %)	169.4 ± 20.3	139.8 ± 5.4		
Fat (g/day)	48.4 ± 1.5	45.9 ± 12.8	2.5	5.4
Vitamin A (μg/day)	1359.0 ± 380.5	1188.5 ± 733.0	170.5	14.3
Vitamin A (DRI %)	339.7 ± 95.1	294.1 ± 183.3		
Vitamin D (μg/day)	19.5 ± 0.1	2.4 ± 0.4	17.1	712.5
Vitamin D (DRI %)	130.0 ± 0.2	16.2 ± 2.5		
Vitamin E (mg/day)	15.3 ± 2.1	15.2 ± 2.9	0.1	0.7
Vitamin E (DRI %)	218.4 ± 29.6	217.4 ± 41.8		
Total folate (μg/day)	243.1 ± 13.9	196.2 ± 78.6	46.9	23.9
Total folate (DRI %)	121.5 ± 7.0	98.1 ± 39.3		
Vitamin B_12_ (μg/day)	2.0 ± 0.1	0.8 ± 0.1	1.2	150.0
Vitamin B_12_ (DRI %)	166.7 ± 0.1	67.5 ± 6.6		
Vitamin C (mg/day)	163.7 ± 25.8	168.8 ± 31.8	5.1	3.0
Vitamin C (DRI %)	654.7 ± 103.1	675.3 ± 127.1		
Sodium (mg/day)	1615.9 ± 752.1	1016.2 ± 174.3	599.7	59.0
Sodium (DRI %)	161.6 ± 75.2	101.6 ± 17.4		
Potassium (mg/day)	2563.7 ± 195.5	1974.0 ± 418.9	589.7	29.9
Potassium (DRI %)	111.5 ± 8.5	85.8 ± 18.2		
Calcium (mg/day)	1415.7 ± 33.4	448.5 ± 98.7	967.2	215.7
Calcium (DRI %)	141.6 ± 3.3	44.8 ± 9.9		
Magnesium (mg/day)	313.3 ± 24.4	155.8 ± 24.7	157.5	101.1
Magnesium (DRI %)	241.0 ± 18.8	119.8 ± 19.0		
Phosphorus (mg/day)	852.7 ± 33.3	540.8 ± 108.4	311.9	57.7
Phosphorus (DRI %)	170.5 ± 6.7	108.2 ± 21.7		
Iron (mg/day)	22.5 ± 0.5	8.6 ± 1.6	13.9	161.6
Iron (DRI %)	224.9 ± 5.4	86.0 ± 15.7		
Zinc (mg/day)	15.3 ± 0.4	6.1 ± 0.9	9.2	150.8
Zinc (DRI %)	306.0 ± 8.4	122.1 ± 17.0		
Dietary fiber (g/day)	13.5 ± 2.4	15.8 ± 2.0	2.3	14.6

**Figure 1:**
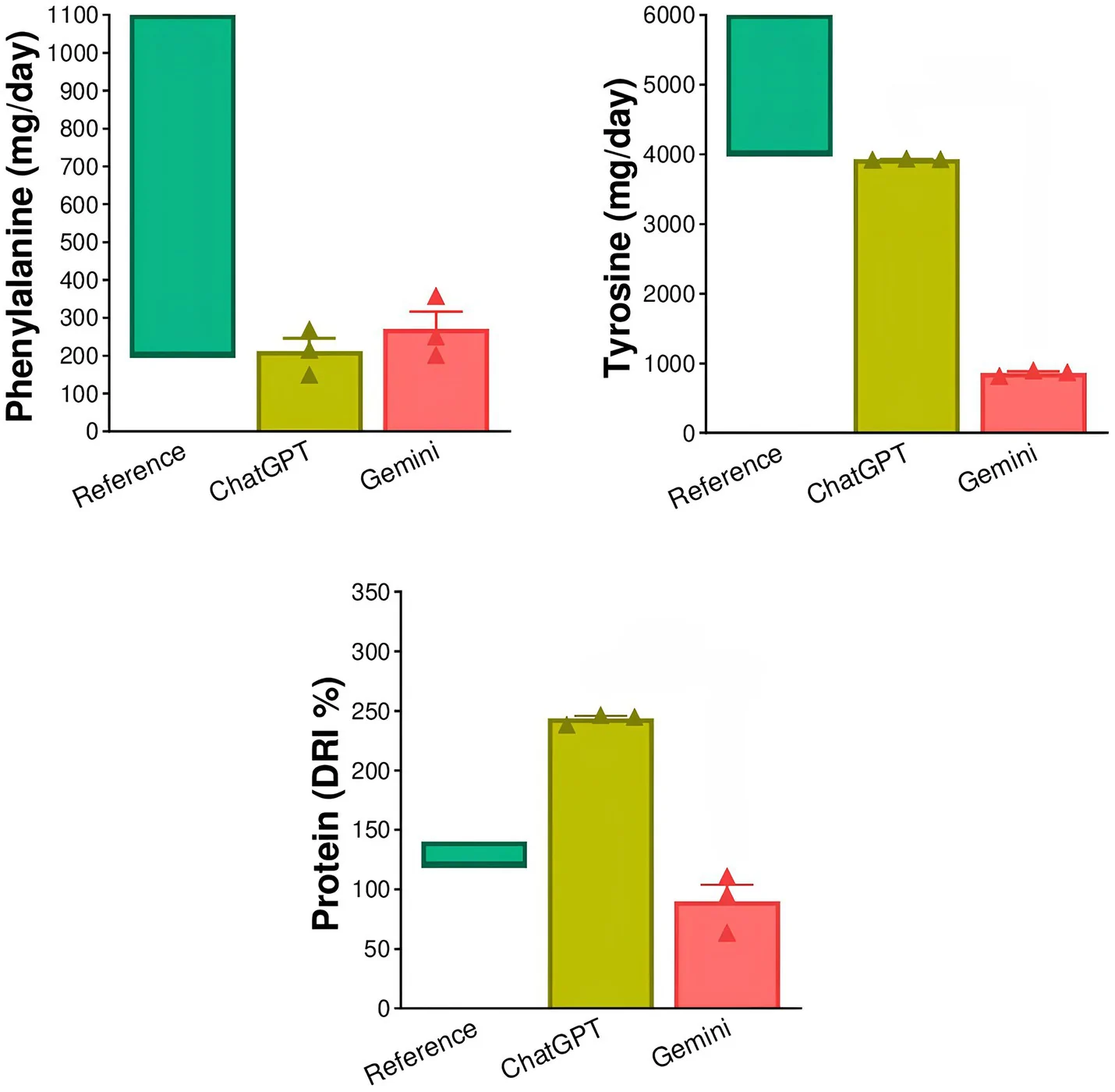
Comparison of protein DRI% levels, phenylalanine, and tyrosine amounts of three-day diet plans generated by ChatGPT and Gemini with the recommended ranges in the GMDI PKU Nutrition Management Guidelines.

Disease-specific benchmarking against the GMDI PKU Nutrition Management Guidelines is presented in Figure 1. Both AI-generated dietary plans provided phenylalanine intakes within the recommended range of 200–1,100 mg/day. In contrast, tyrosine intake remained below the recommended range of 4,000–6,000 mg/day in both dietary plans, with the ChatGPT-generated plan approaching the lower limit (3931.7 ± 9.2 mg/day), whereas the Gemini-generated plan provided substantially lower tyrosine levels (862.8 ± 40.8 mg/day). Protein intake also deviated from the recommended target of 120–140% of the age-specific DRI, with ChatGPT providing 243.3 ± 4.3% and Gemini 90.0 ± 24.2%. Overall, although both AI-generated dietary plans met the recommended phenylalanine target, neither fully complied with all PKU-specific nutritional recommendations.

The amino acid profiles of the AI-generated dietary plans are presented in Table 4. Compared with Gemini, the ChatGPT-generated dietary plan contained higher mean amounts of most amino acids, including leucine, isoleucine, valine, tryptophan, threonine, methionine, alanine, arginine, histidine, lysine, glycine, serine, and proline. Relative differences between the two models were particularly pronounced for arginine, histidine, glycine, proline, and tryptophan, whereas cysteine was the only amino acid present at a higher level in the Gemini-generated dietary plan. Overall, the amino acid profiles demonstrated distinct nutritional patterns between the two AI-generated dietary plans, with ChatGPT consistently providing higher amounts of most amino acids, while Gemini generated lower overall amino acid levels accompanied by markedly lower tyrosine intake. These findings were consistent with the disease-specific benchmarking results for the PKU case.

**Table 4:** Exploratory comparison of daily amino acid contents of 3-day diet plans generated by ChatGPT and Gemini for the PKU case.

	ChatGPT	Gemini	Absolute difference	Relative difference (%)
Leucine (mg/day)	4573.8 ± 84.0	1318.6 ± 163.5	3255.2	246.9
Isoleucine (mg/day)	2719.5 ± 65.6	780.6 ± 107.6	1938.9	284.4
Valine (mg/day)	3026.6 ± 48.1	875.5 ± 139.8	2151.1	245.7
Tryptophan (mg/day)	910.4 ± 14.2	205.7 ± 39.9	704.7	342.6
Threonine (mg/day)	2278.2 ± 29.7	566.6 ± 113.3	1711.6	302.1
Cysteine (mg/day)	51.6 ± 6.2	176.0 ± 28.5	124.4	70.7
Methionine (mg/day)	798.0 ± 8.5	258.1 ± 50.4	539.9	209.2
Alanine (mg/day)	2609.5 ± 41.0	831.4 ± 182.2	1778.1	213.9
Arginine (mg/day)	4262.1 ± 51.5	758.8 ± 155.4	3503.3	461.7
Histidine (mg/day)	1712.5 ± 27.8	299.6 ± 61.2	1412.9	471.6
Lysine (mg/day)	3532.3 ± 72.7	911.2 ± 164.5	2621.1	287.7
Glycine (mg/day)	3969.3 ± 63.4	547.6 ± 127.1	3421.7	624.9
Serine (mg/day)	2429.3 ± 26.5	614.6 ± 178.6	1814.7	295.3
Proline (mg/day)	4204.7 ± 53.6	922.0 ± 186.1	3282.7	355.8

### Maple syrup urine disease (MSUD)

The nutritional characteristics of the AI-generated 3-day dietary plans for the standardized MSUD case are summarized in Table 5. Compared with Gemini, the ChatGPT-generated dietary plan provided a substantially higher mean daily energy intake (1684.5 ± 52.5 vs. 958.2 ± 164.0 kcal/day), representing an absolute difference of 726.3 kcal/day (75.8%). Carbohydrate intake was also considerably higher in the ChatGPT-generated dietary plan, with an absolute difference of 160.0 g/day (127.9%). Among the micronutrients, the largest relative differences were observed for vitamin C, vitamin A, potassium, dietary fiber, total folate, magnesium, and iron, all of which were higher in the ChatGPT-generated dietary plan. In contrast, protein, fat, vitamin D, vitamin E, vitamin B12, sodium, calcium, phosphorus, and zinc showed comparatively smaller differences between the two models. Overall, the ChatGPT-generated dietary plan provided higher energy and carbohydrate levels together with a more nutrient-dense micronutrient profile than the Gemini-generated dietary plan.

**Table 5:** Exploratory comparison of daily energy, macronutrient, micronutrient, and DRI% values of 3-day diet plans generated by ChatGPT and Gemini for the MSUD case.

	ChatGPT	Gemini	Absolute difference	Relative difference (%)
Energy (kcal/day)	1684.5 ± 52.5	958.2 ± 164.0	726.3	75.8
Protein (g/day)	25.7 ± 2.5	21.9 ± 2.3	3.8	17.4
Protein (DRI %)	135.3 ± 12.9	115.2 ± 12.0		
Carbohydrate (g/day)	285.1 ± 13.3	125.1 ± 11.9	160.0	127.9
Carbohydrate (DRI %)	219.3 ± 10.2	96.2 ± 9.2		
Fat (g/day)	44.2 ± 1.2	38.7 ± 12.8	5.5	14.2
Vitamin A (μg/day)	1204.7 ± 19.1	450.9 ± 188.9	753.8	167.2
Vitamin A (DRI %)	301.2 ± 4.8	112.7 ± 47.2		
Vitamin D (μg/day)	13.1 ± 0.1	13.3 ± 0.2	0.2	1.5
Vitamin D (DRI %)	87.2 ± 1.0	88.7 ± 1.0		
Vitamin E (mg/day)	13.8 ± 2.1	10.8 ± 3.8	3.0	27.8
Vitamin E (DRI %)	197.2 ± 29.6	154.1 ± 54.2		
Total folate (μg/day)	294.6 ± 26.1	187.7 ± 33.2	106.9	56.9
Total folate (DRI %)	147.3 ± 13.0	93.9 ± 16.6		
Vitamin B_12_ (μg/day)	0.8 ± 0.1	0.8 ± 0.2	0	0
Vitamin B_12_ (DRI %)	65.0 ± 0.1	69.7 ± 8.2		
Vitamin C (mg/day)	220.7 ± 8.0	71.2 ± 10.3	149.5	209.9
Vitamin C (DRI %)	882.8 ± 31.9	285.0 ± 41.0		
Sodium (mg/day)	932.0 ± 298.8	742.8 ± 205.2	189.2	25.5
Sodium (DRI %)	93.2 ± 29.9	74.3 ± 20.5		
Potassium (mg/day)	2152.6 ± 223.3	1075.7 ± 173.6	1076.9	100.1
Potassium (DRI %)	93.6 ± 9.7	46.8 ± 7.5		
Calcium (mg/day)	804.6 ± 21.6	717.5 ± 25.8	87.1	12.1
Calcium (DRI %)	80.5 ± 2.2	71.7 ± 2.6		
Magnesium (mg/day)	199.5 ± 29.3	132.7 ± 20.3	66.8	50.3
Magnesium (DRI %)	153.5 ± 22.6	102.1 ± 15.6		
Phosphorus (mg/day)	703.0 ± 57.1	610.4 ± 104.4	92.6	15.2
Phosphorus (DRI %)	140.6 ± 11.4	122.1 ± 20.9		
Iron (mg/day)	11.3 ± 0.9	8.5 ± 0.5	2.8	33.0
Iron (DRI %)	112.9 ± 8.8	84.9 ± 4.6		
Zinc (mg/day)	5.4 ± 0.8	4.4 ± 0.6	1.0	22.7
Zinc (DRI %)	107.1 ± 15.7	87.6 ± 11.2		
Dietary fiber (g/day)	24.2 ± 1.4	13.7 ± 1.5	10.5	76.6

**Figure 2:**
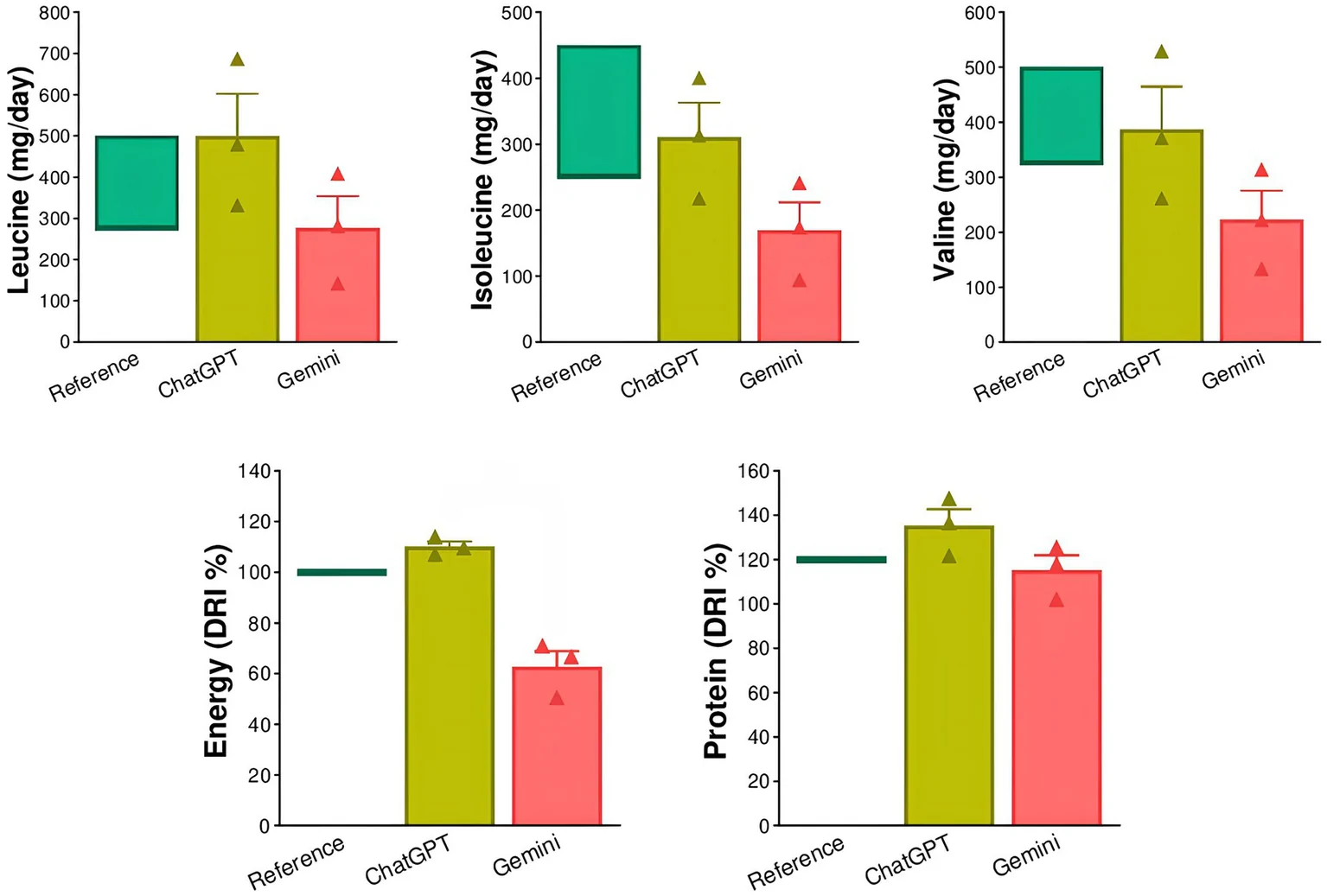
Comparison of energy DRI% and protein DRI% levels, and branched-chain amino acid (leucine, isoleucine, and valine) amounts of three-day diet plans generated by ChatGPT and Gemini with the recommended ranges in the GMDI MSUD Nutrition Management Guidelines.

Disease-specific benchmarking according to the GMDI MSUD Nutrition Management Guidelines is presented in Figure 2. The ChatGPT-generated dietary plan provided energy intake equivalent to 110.2 ± 3.5% of the age-specific DRI, whereas the Gemini-generated plan provided 62.7 ± 10.7%, indicating substantially lower energy provision. Protein intake was closer to the recommended target of 120% of the DRI in both dietary plans, with ChatGPT providing 135.3 ± 12.9% and Gemini 115.2 ± 12.0%. With respect to the branched-chain amino acids, both models generated leucine values within the recommended range, whereas isoleucine and valine remained below the recommended ranges in the Gemini-generated dietary plan. Overall, the ChatGPT-generated dietary plan more closely approximated the GMDI recommendations for energy provision and branched-chain amino acid targets; however, neither AI-generated dietary plan fully complied with all disease-specific nutritional recommendations for MSUD.

The amino acid profiles of the AI-generated dietary plans are presented in Table 6. Compared with Gemini, the ChatGPT-generated dietary plan consistently provided higher mean amounts of phenylalanine, tyrosine, tryptophan, threonine, methionine, alanine, arginine, histidine, lysine, glycine, serine, and proline. However, the relative differences between the two models were generally modest, ranging from 3.7 to 17.3% for all amino acids. The largest relative differences were observed for serine, arginine, histidine, and lysine, whereas cysteine showed the smallest difference between the two dietary plans. Overall, the amino acid profiles generated by the two AI models were broadly comparable, indicating that the principal differences between the dietary plans for the MSUD case were related to overall dietary composition and energy provision rather than substantial variation in amino acid distribution.

**Table 6:** Exploratory comparison of daily amino acid contents of 3-day diet plans generated by ChatGPT and Gemini for the MSUD case.

	ChatGPT	Gemini	Absolute difference	Relative difference (%)
Phenylalanine (mg/day)	1438.0 ± 100.5	1290.0 ± 67.5	148.0	11.5
Tyrosine (mg/day)	1746.2 ± 81.2	1638.0 ± 25.4	108.2	6.6
Tryptophan (mg/day)	426.2 ± 32.3	383.3 ± 16.8	42.9	11.2
Threonine (mg/day)	1866.7 ± 92.1	1741.9 ± 63.4	124.8	7.2
Cysteine (mg/day)	1216.1 ± 45.7	1172.5 ± 21.4	43.6	3.7
Methionine (mg/day)	585.9 ± 54.7	527.6 ± 29.1	58.3	11.1
Alanine (mg/day)	2912.7 ± 151.5	2741.3 ± 121.1	171.4	6.3
Arginine (mg/day)	1372.3 ± 151.0	1205.2 ± 115.6	167.1	13.9
Histidine (mg/day)	715.0 ± 55.1	636.7 ± 45.1	78.3	12.3
Lysine (mg/day)	1699.1 ± 103.3	1517.3 ± 69.0	181.8	12.0
Glycine (mg/day)	2572.0 ± 96.8	2441.6 ± 88.1	130.4	5.3
Serine (mg/day)	965.1 ± 115.3	823.1 ± 97.2	142.0	17.3
Proline (mg/day)	1577.3 ± 149.7	1429.8 ± 107.3	147.5	10.3

### Propionic acidemia (PPA)

The nutritional characteristics of the AI-generated 3-day dietary plans for the standardized PPA case are summarized in Table 7. Compared with Gemini, the ChatGPT-generated dietary plan provided a higher mean daily energy intake (1662.0 ± 19.2 vs. 1401.0 ± 38.7 kcal/day), corresponding to an absolute difference of 261.0 kcal/day (18.6%). Carbohydrate intake was also higher in the ChatGPT-generated dietary plan, with an absolute difference of 66.3 g/day (37.5%). Among the micronutrients, the largest relative difference was observed for vitamin A, which was higher in the ChatGPT-generated dietary plan. In contrast, differences between the two models were comparatively modest for fat, vitamin E, total folate, vitamin C, potassium, magnesium, phosphorus, iron, zinc, and dietary fiber, while Gemini generated higher amounts of vitamin D, vitamin B12, sodium, and calcium. Overall, the two AI-generated dietary plans demonstrated broadly comparable micronutrient profiles despite differences in energy provision and carbohydrate content.

**Table 7:** Exploratory comparison of daily energy, macronutrient, micronutrient, and DRI% values of 3-day diet plans generated by ChatGPT and Gemini for the PPA case.

	ChatGPT	Gemini	Absolute difference	Relative difference (%)
Energy (kcal/day)	1662.0 ± 19.2	1401.0 ± 38.7	261.0	18.6
Protein (g/day)	39.2 ± 2.8	45.7 ± 8.1	6.5	14.2
Protein (DRI %)	206.4 ± 14.8	240.7 ± 42.5		
Carbohydrate (g/day)	243.1 ± 5.2	176.8 ± 1.4	66.3	37.5
Carbohydrate (DRI %)	187.0 ± 4.0	136.0 ± 1.1		
Fat (g/day)	58.8 ± 1.5	58.0 ± 3.2	0.8	1.4
Vitamin A (μg/day)	1733.3 ± 168.0	1088.0 ± 121.7	645.3	59.3
Vitamin A (DRI %)	433.3 ± 42.0	272.0 ± 30.4		
Vitamin D (μg/day)	28.0 ± 0.1	36.0 ± 8.1	8.0	22.2
Vitamin D (DRI %)	186.3 ± 0.2	240.2 ± 54.2		
Vitamin E (mg/day)	20.1 ± 0.9	21.5 ± 3.7	1.4	6.5
Vitamin E (DRI %)	286.4 ± 13.1	307.0 ± 53.0		
Total folate (μg/day)	410.0 ± 41.9	451.7 ± 59.3	41.7	9.2
Total folate (DRI %)	205.0 ± 21.0	225.9 ± 29.6		
Vitamin B_12_ (μg/day)	1.7 ± 0.1	2.2 ± 0.5	0.5	22.7
Vitamin B_12_ (DRI %)	142.2 ± 0.5	183.6 ± 42.2		
Vitamin C (mg/day)	155.0 ± 41.1	155.0 ± 32.0	0	0
Vitamin C (DRI %)	620.1 ± 164.6	620.1 ± 128.0		
Sodium (mg/day)	1170.6 ± 398.8	1551.6 ± 293.8	381.0	24.6
Sodium (DRI %)	117.1 ± 39.9	155.2 ± 29.4		
Potassium (mg/day)	2167.5 ± 508.4	1912.7 ± 222.3	254.8	13.3
Potassium (DRI %)	94.2 ± 22.1	83.2 ± 9.7		
Calcium (mg/day)	1453.3 ± 19.1	1776.3 ± 336.9	323.0	18.2
Calcium (DRI %)	145.3 ± 1.9	177.6 ± 33.7		
Magnesium (mg/day)	253.6 ± 44.1	267.3 ± 29.1	13.7	5.1
Magnesium (DRI %)	195.1 ± 33.9	205.6 ± 22.4		
Phosphorus (mg/day)	1094.0 ± 56.9	1279.4 ± 219.8	185.4	14.5
Phosphorus (DRI %)	218.8 ± 11.4	255.9 ± 44.0		
Iron (mg/day)	17.4 ± 1.2	20.5 ± 2.2	3.1	15.1
Iron (DRI %)	174.4 ± 11.9	205.3 ± 22.4		
Zinc (mg/day)	8.2 ± 0.8	10.0 ± 1.9	1.8	18.0
Zinc (DRI %)	164.7 ± 16.4	200.9 ± 38.0		
Dietary fiber (g/day)	24.7 ± 1.5	25.4 ± 2.0	0.7	2.8

**Figure 3:**
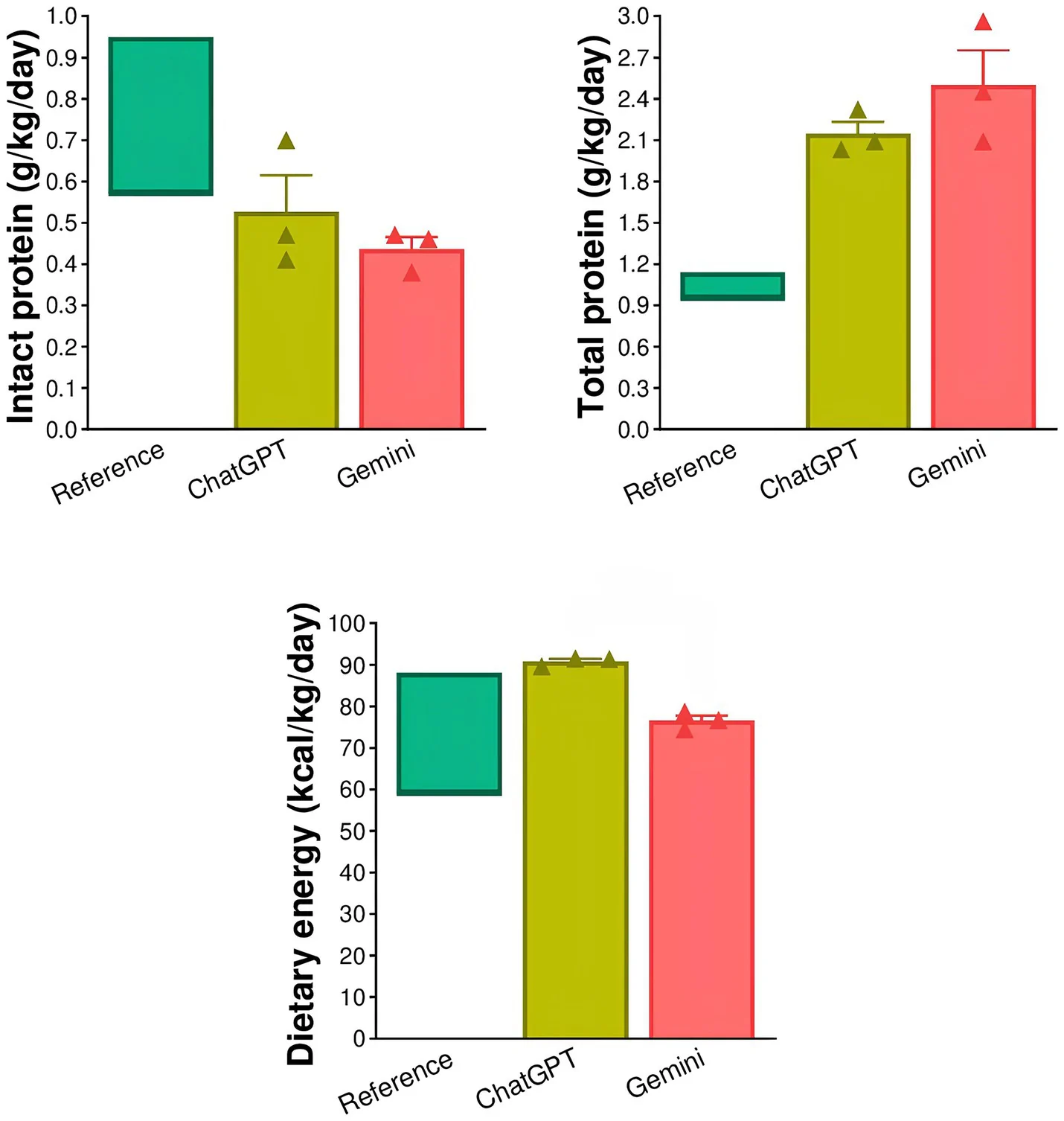
Comparison of intact protein, total protein, and dietary energy levels of three-day diet plans generated by ChatGPT and Gemini with the recommended ranges in the GMDI PPA Nutrition Management Guidelines.

Disease-specific benchmarking according to the GMDI PPA Nutrition Management Guidelines is presented in Figure 3. In both AI-generated dietary plans, intact protein intake remained below the recommended range of 0.57–0.95 g/kg/day, with mean values of 0.53 ± 0.15 g/kg/day for ChatGPT and 0.44 ± 0.05 g/kg/day for Gemini. Conversely, total protein intake exceeded the recommended range of 0.95–1.14 g/kg/day in both dietary plans, reaching 2.15 ± 0.15 g/kg/day and 2.50 ± 0.44 g/kg/day for ChatGPT and Gemini, respectively. Dietary energy provision differed between the two models: the ChatGPT-generated dietary plan slightly exceeded the upper recommended limit (90.8 ± 1.1 kcal/kg/day), whereas the Gemini-generated dietary plan remained within the recommended range (76.6 ± 2.1 kcal/kg/day). Overall, neither AI-generated dietary plan fully complied with the disease-specific nutritional targets for PPA.

The amino acid profiles of the AI-generated dietary plans are presented in Table 8. Gemini-generated dietary plan generally provided higher mean amounts of leucine, phenylalanine, tyrosine, tryptophan, cysteine, alanine, arginine, histidine, lysine, glycine, serine, and proline, whereas ChatGPT generated higher amounts of isoleucine, valine, threonine, and methionine. The relative differences between the two models were greatest for isoleucine, valine, threonine, and methionine, while differences for the remaining amino acids were generally modest. Overall, the amino acid profiles generated by the two AI models were broadly comparable despite differences in the relative distribution of individual amino acids. Notably, the propionate precursor amino acids (isoleucine, valine, methionine, and threonine), which are of particular clinical relevance in PPA, showed differences in their relative distribution between the two dietary plans.

**Table 8:** Exploratory comparison of daily amino acid contents of 3-day diet plans generated by ChatGPT and Gemini for the PPA case.

	ChatGPT	Gemini	Absolute difference	Relative difference (%)
Leucine (mg/day)	5405.8 ± 192.0	6497.8 ± 1294.0	1092.0	16.8
Isoleucine (mg/day)	431.7 ± 118.4	261.8 ± 51.2	169.9	64.9
Valine (mg/day)	482.0 ± 201.3	287.9 ± 89.5	194.1	67.4
Phenylalanine (mg/day)	2487.1 ± 128.8	2912.6 ± 555.6	425.5	14.6
Tyrosine (mg/day)	2391.3 ± 81.7	2837.1 ± 542.9	445.8	15.7
Tryptophan (mg/day)	840.0 ± 50.0	995.9 ± 187.7	155.9	15.7
Threonine (mg/day)	320.6 ± 131.1	195.7 ± 60.5	124.9	63.8
Cysteine (mg/day)	1751.9 ± 35.7	2179.2 ± 461.2	427.3	19.6
Methionine (mg/day)	151.5 ± 81.6	102.6 ± 36.6	48.9	47.7
Alanine (mg/day)	4072.9 ± 184.2	4901.2 ± 989.3	828.3	16.9
Arginine (mg/day)	3615.9 ± 214.1	4342.6 ± 854.7	726.7	16.7
Histidine (mg/day)	2331.9 ± 89.4	2838.8 ± 584.1	506.9	17.9
Lysine (mg/day)	3703.7 ± 227.8	4393.7 ± 828.1	690.0	15.7
Glycine (mg/day)	1824.0 ± 160.0	2076.8 ± 394.0	252.8	12.2
Serine (mg/day)	2524.0 ± 175.1	2949.2 ± 589.2	425.2	14.4
Proline (mg/day)	1888.0 ± 112.7	2185.3 ± 531.4	297.3	13.6

## Discussion

The present study provides an exploratory benchmarking analysis of AI-generated dietary plans for three inherited protein metabolism disorders requiring highly specialized nutritional management. Using standardized pediatric case scenarios and identical prompting conditions, the nutritional outputs generated by two widely used large language models were evaluated against both general dietary reference standards and disease-specific recommendations published by Genetic Metabolic Dietitians International (GMDI). Overall, the findings demonstrated that although both AI models were capable of generating structured dietary plans with acceptable nutritional characteristics for several macro- and micronutrients, neither consistently achieved all disorder-specific nutritional targets required for the dietary management of PKU, MSUD, and PPA. Importantly, the observed deviations differed not only between AI models but also according to the metabolic disorder being evaluated, highlighting the complexity of applying general-purpose LLMs to highly specialized areas of clinical nutrition. Rather than identifying a universally superior AI model, the present findings emphasize that the clinical suitability of AI-generated dietary plans should be judged according to disease-specific nutritional objectives and metabolic safety considerations. These findings extend the growing literature on AI-assisted nutrition planning by demonstrating that conventional measures of nutritional adequacy alone are insufficient for evaluating dietary recommendations in inherited metabolic disorders, where precise control of specific amino acids, protein sources, and medical nutrition therapy is essential.

Inherited protein metabolism disorders represent one of the most challenging areas of clinical nutrition because dietary treatment requires not only adequate energy and nutrient intake but also precise regulation of disease-specific amino acids and appropriate integration of medical nutrition products (2). Unlike conventional dietary planning, nutritional management in disorders such as PKU, MSUD, and PPA aims to maintain metabolic stability by balancing natural protein intake, specialized amino acid formulas, and individual amino acid tolerance while simultaneously supporting normal growth and development (26, 32). Even relatively small deviations from recommended dietary targets may result in metabolic decompensation, neurocognitive impairment, or long-term clinical complications, particularly in pediatric patients, where nutritional requirements continuously change with age and growth (1, 26, 33). Consequently, evaluating AI-generated dietary plans for inherited metabolic disorders requires a different perspective from that used in studies of general nutrition. In this context, compliance with disease-specific nutritional recommendations and metabolic safety thresholds is considerably more informative than assessment based solely on overall nutrient adequacy or adherence to Dietary Reference Intakes.

The findings for the PKU case highlight one of the principal challenges in AI-assisted dietary planning for inherited metabolic disorders: achieving an appropriate balance between adequate protein provision and strict amino acid control. Although both AI-generated dietary plans maintained phenylalanine intake within the recommended range, neither model simultaneously achieved the recommended targets for protein and tyrosine intake. ChatGPT generated substantially higher total protein intake than recommended, whereas the Gemini-generated dietary plans were characterized by insufficient protein and markedly lower tyrosine intake. These findings are clinically important because successful dietary management of PKU depends not only on restricting phenylalanine but also on providing sufficient protein equivalents and tyrosine to support normal growth, neurodevelopment, and protein synthesis (26, 34). Since tyrosine becomes a conditionally essential amino acid in PKU owing to impaired phenylalanine hydroxylation, inadequate tyrosine intake may contribute to suboptimal neurotransmitter synthesis and impaired metabolic control despite apparently acceptable phenylalanine intake (26, 34). Therefore, the present findings suggest that evaluation of AI-generated dietary plans for PKU should extend beyond phenylalanine restriction alone and include comprehensive assessment of protein quality, protein equivalents supplied by medical formulas, and tyrosine adequacy.

The findings obtained for the MSUD case further demonstrate that evaluating AI-generated dietary plans for inherited metabolic disorders requires consideration of overall metabolic stability rather than individual nutrient values alone. Although the two AI-generated dietary plans showed broadly comparable amino acid profiles, important differences were observed in overall dietary composition, particularly with respect to energy provision. Disease-specific benchmarking indicated that the ChatGPT-generated dietary plans more closely approximated the recommended energy intake, whereas the Gemini-generated dietary plans provided considerably lower energy levels. Similarly, the branched-chain amino acid (BCAA) values generated by ChatGPT generally showed closer agreement with the recommended ranges than those generated by Gemini, although neither model consistently fulfilled all disease-specific nutritional targets.

These findings are clinically relevant because dietary management of MSUD extends beyond dietary leucine restriction and requires adequate energy intake to suppress endogenous protein catabolism and maintain metabolic homeostasis (8, 9). During periods of inadequate energy intake, increased proteolysis may elevate circulating leucine and other BCAAs despite appropriate dietary restriction, thereby increasing the risk of metabolic decompensation (8). Consequently, successful nutritional management depends on simultaneously achieving appropriate energy intake, controlled BCAA exposure, and sufficient protein equivalents from medical nutrition therapy rather than focusing on a single dietary parameter.

These findings suggest that current general-purpose LLMs can generate nutritionally plausible dietary plans but remain limited in simultaneously addressing the complex and interdependent nutritional requirements of inherited metabolic disorders such as MSUD. Recent reviews have emphasized that AI offers substantial opportunities to support nutrition practice while also highlighting the need for careful human oversight, particularly when dietary recommendations require individualized clinical decision-making and disease-specific expertise (35, 36). Therefore, AI-generated dietary plans for inherited metabolic disorders should be regarded as decision-support tools rather than autonomous clinical recommendations and should always be reviewed by experienced metabolic dietitians before implementation.

The findings obtained for the PPA case highlight an important limitation of current general-purpose LLMs in metabolic nutrition planning: the difficulty of appropriately balancing intact protein, total protein, and dietary energy. Although both AI-generated dietary plans provided broadly comparable amino acid profiles, disease-specific benchmarking revealed that neither model achieved the recommended distribution of protein sources. In both dietary plans, intact protein intake remained below the recommended range, whereas total protein intake substantially exceeded the guideline recommendations. These findings indicate that overall protein provision alone is not an adequate indicator of nutritional suitability in PPA.

The distinction between intact protein and total protein is fundamental in the dietary management of propionic acidemia because natural protein supplies propiogenic amino acids that must be carefully controlled, whereas total protein includes protein equivalents provided by precursor-free medical formulas (11, 32). Achieving an appropriate balance between these two protein sources is essential to support normal growth while minimizing the metabolic burden associated with propiogenic amino acids. Therefore, evaluation of dietary plans based solely on total protein intake may overlook clinically important deviations in natural protein provision.

The present findings suggest that although AI-generated dietary plans may estimate overall protein provision, they may have limited capacity to appropriately integrate the complex relationship between intact protein, medical nutrition therapy, and disorder-specific metabolic targets. This observation is particularly relevant because dietary management of PPA requires continuous adjustment of natural protein tolerance according to metabolic control, age, growth, biochemical monitoring, and clinical status (11, 32). These findings reinforce the importance of expert clinical supervision when interpreting AI-generated dietary plans for PPA, particularly where individualized adjustment of medical formulas and intact protein intake is required.

The present findings should be interpreted within the context of the rapidly expanding literature on artificial intelligence in nutrition and dietetics. Recent studies have demonstrated that large language models can generate meal plans, provide dietary recommendations, and support nutrition-related decision-making with promising accuracy in a variety of nutritional settings (12, 14, 18, 37). Nevertheless, most published evaluations have assessed AI performance using general measures of nutritional adequacy, dietary quality, or agreement with dietary guidelines. Comparatively fewer studies have examined AI-generated dietary recommendations in clinical conditions requiring highly individualized medical nutrition therapy.

The present study extends this emerging field by evaluating AI-generated dietary plans against disease-specific metabolic nutrition guidelines rather than relying exclusively on conventional nutritional assessment. Across all three inherited protein metabolism disorders, both AI models generated nutritionally structured dietary plans; however, clinically relevant deviations from GMDI recommendations remained evident for critical nutritional targets, including amino acid control, protein distribution, and energy provision. These findings indicate that conventional indicators of nutritional adequacy alone are insufficient for evaluating AI-generated dietary plans in inherited metabolic disorders, where clinical appropriateness depends on precise integration of metabolic requirements, medical nutrition products, and individualized dietary tolerance.

From a clinical perspective, the present findings indicate that AI-generated dietary plans have the potential to assist in developing an initial dietary framework for inherited protein metabolism disorders. Nevertheless, the clinically relevant deviations identified across all three disorders demonstrate that overall nutritional adequacy does not necessarily translate into disease-specific nutritional appropriateness. Accordingly, dietary plans generated by general-purpose LLMs should be evaluated against established metabolic nutrition guidelines before they are considered for clinical application.

The dietary management of inherited protein metabolism disorders requires continuous integration of biochemical monitoring, individual amino acid tolerance, growth assessment, medical nutrition products, and clinical judgment. These complex and dynamic factors cannot be fully incorporated into a single standardized prompt. Consequently, interpretation and individualized adaptation of AI-generated dietary plans remain dependent on multidisciplinary clinical expertise, particularly in disorders where relatively small deviations from guideline recommendations may have important metabolic consequences.

Several methodological strengths should be considered when interpreting the findings of the present study. First, all comparisons were performed using standardized pediatric case scenarios and identical prompts, allowing differences in the generated dietary plans to be attributed primarily to the outputs of the AI models rather than variations in clinical input. Second, dietary plans were evaluated using both general nutritional adequacy (Dietary Reference Intakes) and disease-specific metabolic nutrition guidelines developed by Genetic Metabolic Dietitians International, providing a clinically relevant benchmarking framework beyond conventional nutrient analysis. Third, the assessment included not only energy and macro- and micronutrient composition but also detailed amino acid profiling, enabling evaluation of nutritional characteristics that are directly relevant to the dietary management of inherited protein metabolism disorders. Collectively, these methodological features provide a comprehensive framework for assessing the clinical appropriateness of AI-generated dietary plans in highly specialized areas of metabolic nutrition.

The findings of this study should be interpreted in light of several limitations. First, the evaluation was based on a single standardized pediatric case scenario for each inherited protein metabolism disorder. Although this approach enhanced methodological consistency and enabled direct comparison of AI-generated dietary plans under identical conditions, it does not capture the considerable inter-individual variability encountered in routine clinical practice. Second, dietary plans were generated from a single interaction with each AI model without iterative prompt refinement. This design was intentionally adopted to evaluate the performance of general-purpose LLMs under standardized conditions; however, alternative prompting strategies may have produced different outputs. Third, only two widely used commercial LLMs were evaluated, and their performance may not be representative of other existing or future AI systems. Furthermore, because large language models are continuously updated, their responses may change over time, potentially affecting the reproducibility of AI-generated dietary recommendations. Finally, this was a simulation-based benchmarking study that evaluated nutritional characteristics of AI-generated dietary plans rather than their clinical implementation or patient outcomes. Consequently, future studies should examine the performance of AI-assisted dietary planning using multiple clinical scenarios, repeated prompt strategies, additional AI models, and prospective clinical validation in patients with inherited metabolic disorders.

## Conclusion

This exploratory simulation-based benchmarking study demonstrated that general-purpose large language models are capable of generating structured dietary plans for inherited protein metabolism disorders; however, neither model consistently fulfilled all disease-specific nutritional targets recommended by current metabolic nutrition guidelines. Although the AI-generated dietary plans generally provided acceptable overall nutritional adequacy, clinically relevant deviations were identified in amino acid control, protein distribution, and energy provision across PKU, MSUD, and PPA.

These findings demonstrate that evaluation of AI-generated dietary plans for inherited protein metabolism disorders should extend beyond conventional nutritional assessment and incorporate disease-specific benchmarking against established metabolic nutrition guidelines. Collectively, this study provides a disease-specific benchmarking framework for evaluating AI-generated dietary plans in inherited protein metabolism disorders and contributes to the emerging evidence supporting the responsible integration of artificial intelligence into specialized metabolic nutrition practice.

## Data Availability

The original contributions presented in the study are included in the article/Supplementary material, further inquiries can be directed to the corresponding author.
