# Supplementary material for: Exploratory benchmarking of AI-generated diet plans for inherited protein metabolism disorders: a simulation-based evaluation of nutritional accuracy and clinical safety
Source: Front Nutr. 2026 Aug 26;13:1877144. doi: 10.3389/fnut.2026.1877144 (PMC13556157; doi:10.3389/fnut.2026.1877144)
Supplement: Supplementary file 1 [file Table_1.DOCX]

**Appendix Table 1.** Three-day dietary plan generated by ChatGPT for a pediatric PKU case

| Day | Meal | Food item | Amount/Grammage |
| --- | --- | --- | --- |
| Day 1 | Breakfast | Phe-free amino acid formula* | 200 ml |
|  |  | Low-protein bread | 30 g |
|  |  | Sugar-free jam | 15 g |
|  |  | Cucumber | 50 g |
|  |  | Olive oil | 5 ml |
|  | Morning snack | Apple | 120 g |
|  |  | Low-protein rice cakes | 14 g |
|  | Lunch | Low-protein pasta (cooked) | 150 g |
|  |  | Tomato sauce (tomato + onion) (60 + 20 g) | 80 g |
|  |  | Olive oil | 10 ml |
|  |  | Lettuce | 50 g |
|  |  | Lemon juice | 10 g |
|  | Afternoon snack | Phe-free amino acid formula* | 150 ml |
|  |  | Pear | 100 g |
|  | Dinner | Vegetable soup (zucchini + carrot + potato) (50 + 30 + 40 g) | 250 ml |
|  |  | Low-protein bread | 30 g |
|  |  | Olive oil | 5 ml |
|  | Evening snack | Phe-free amino acid formula* | 150 ml |
| Day 2 | Breakfast | Phe-free amino acid formula* | 200 ml |
|  |  | Low-protein toast bread | 30 g |
|  |  | Honey | 10 g |
|  |  | Banana | 60 g |
|  | Morning snack | Mandarin | 120 g |
|  |  | Low-protein crackers | 20 g |
|  | Lunch | Low-protein rice (cooked) | 160 g |
|  |  | Stewed vegetables (eggplant + zucchini + pepper) (40 + 40 + 30 g) | 110 g |
|  |  | Olive oil | 10 ml |
|  | Afternoon snack | Phe-free amino acid formula* | 150 ml |
|  |  | Apple juice (unsweetened) | 120 ml |
|  | Dinner | Mashed potatoes | 120 g |
|  |  | Olive oil | 10 ml |
|  |  | Low-protein bread | 30 g |
|  |  | Tomato | 50 g |
|  | Evening snack | Phe-free amino acid formula* | 150 ml |
| Day 3 | Breakfast | Phe-free amino acid formula* | 200 ml |
|  |  | Low-protein pancake (LP flour) | 40 g |
|  |  | Sugar | 5 g |
|  |  | Vegetable oil | 5 g |
|  |  | Strawberry puree | 50 g |
|  | Morning snack | Grapes | 100 g |
|  |  | Low-protein biscuits | 20 g |
|  | Lunch | Low-protein spaghetti (cooked) | 150 g |
|  |  | Tomato sauce (tomato + onion) (60 + 20 g) | 80 g |
|  |  | Olive oil | 10 ml |
|  | Afternoon Snack | Phe-free amino acid formula* | 150 ml |
|  |  | Peach | 120 g |
|  | Dinner | Vegetable stir-fry (zucchini + carrot) (60 + 40 g) | 100 g |
|  |  | Low-protein rice (cooked) | 120 g |
|  |  | Olive oil | 10 ml |
|  | Evening snack | Phe-free amino acid formula* | 150 ml |

*Nutricia PKU Anamix Junior LQ ®

**Appendix Table 2.** Three-day dietary plan generated by Gemini for a pediatric PKU case

| Day | Meal | Food item | Amount/Grammage |
| --- | --- | --- | --- |
| Day 1 | Breakfast | Low-protein bread (toasted) | 2 slices (60 g) |
|  |  | Honey or jam | 1 tbsp (20 g) |
|  |  | Sliced cucumber & tomato | 50 g each |
|  |  | Medical formula* | 1 dose |
|  | Lunch | Low-protein with tomato sauce | 60 g (dry weight) |
|  |  | Sauteed zucchini & peppers | 80 g |
|  |  | Apple | 1 small (100 g) |
|  | Afternoon snack | Low-protein crackers | 3-4 pieces (20 g) |
|  |  | Pear | 1/2 medium (80 g) |
|  | Dinner | Vegetable soup (zucchini + potato + onion + sunflower oil) (25 + 15 + 20 + 5 g) | 1 bowl (200 ml) |
|  |  | Low-protein bread | 1 slice (30 g) |
|  |  | Roasted carrots | 70 g |
|  |  | Medical formula* | 1 dose |
| Day 2 | Breakfast | Low-protein pancakes (with low-protein flour) | 2 small (80 g) |
|  |  | Maple syrup | 1 tbsp (15 g) |
|  |  | Strawberries | 5-6 berries (100 g) |
|  |  | Medical formula* | 1 dose |
|  | Lunch | Low-protein veggie burger (low-protein bun + mushroom patty) | 1 unit (120 g) |
|  |  | Homemade potato wedges | 80 g |
|  |  | Lettuce & onion garnish | 30 g |
|  | Afternoon snack | Low-protein bar | 1 bar (30g) |
|  |  | Grapes | 10 small (60g) |
|  | Dinner | Cauliflower "rice" with turmeric | 150 g |
|  |  | Stir-fried green beans | 100 g |
|  |  | Medical formula* | 1 dose |
| Day 3 | Breakfast | Low-protein porridge (with low-protein milk sub) | 1 bowl (150 g) |
|  |  | Blueberries | 50 g |
|  |  | Medical formula | 1 dose |
|  | Lunch | Low-protein vegetable pizza (tomato sauce + mozzarella cheese substitute + sweet peppers + button mushrooms + boiled corn + olive oil) (15 + 15 + 5 + 5 + 5 + 5 g) | 1 medium slice (100 g) |
|  |  | Mixed green salad (lettuce + carrots + red cabbage + olive oil) (55 + 20 + 15 + 10 g) | 100 g |
|  |  | Orange | 1 small (100 g) |
|  | Afternoon Snack | Low-protein pretzels | 15 g |
|  |  | Watermelon | 100 g |
|  | Dinner | Low-protein couscous with roasted eggplant | 50 g (dry) / 100 g eggplant |
|  |  | Sautéed spinach | 80 g |
|  |  | Medical formula* | 1 dose |

*Phenyl-free 2 ® (powder product – 1 dose: 15 g)

**Appendix Table 3.** Three-day dietary plan generated by ChatGPT for a pediatric MSUD case

| Day | Meal | Food item | Amount/Grammage |
| --- | --- | --- | --- |
| Day 1 | Breakfast | Low-protein bread | 40 g |
|  |  | Apple jam | 15 g |
|  |  | Dairy-free margarine | 5 g |
|  |  | Cucumber | 30 g |
|  |  | Fresh orange juice | 150 ml |
|  |  | MSUD formula* | 20 g powder |
|  | Morning snack | Banana | 80 g |
|  |  | Rice cakes (low protein) | 10 g |
|  | Lunch | Low-protein pasta (dry) | 50 g |
|  |  | Tomato | 80 g |
|  |  | Olive oil | 10 ml |
|  |  | Onion | 20 g |
|  |  | Green salad (lettuce + arugula + olive oil) (25+25+5 g) | 55 g |
|  |  | Apple compote | 100 g |
|  | Afternoon snack | Pear | 100 g |
|  |  | Homemade lemonade | 150 ml |
|  | Dinner | Low-protein rice (dry) | 45 g |
|  |  | Zucchini | 60 g |
|  |  | Carrot | 40 g |
|  |  | Potato | 50 g |
|  |  | Olive oil | 10 ml |
|  |  | Low-protein bread | 30 g |
|  |  | MSUD formula* | 20 g powder |
|  | Evening snack | Strawberry jelly dessert | 120 g |
|  |  | MSUD formula* | 20 g powder |
| Day 2 | Breakfast | Low-protein flour | 35 g |
|  |  | Sugar | 10 g |
|  |  | Oil | 5 g |
|  |  | Maple syrup | 10 g |
|  |  | Strawberries | 70 g |
|  |  | MSUD formula* | 20 g powder |
|  | Morning snack | Peach | 100 g |
|  |  | Fruit smoothie (apple juice) | 150 ml |
|  | Lunch | Low-protein flour | 60 g |
|  |  | Tomato sauce | 50 g |
|  |  | Bell pepper | 30 g |
|  |  | Olive oil | 10 ml |
|  |  | Corn | 40 g |
|  |  | Lettuce | 30 g |
|  |  | Olive oil | 5 ml |
|  |  | Grapes | 80 g |
|  | Afternoon snack | Low-protein biscuits | 20 g |
|  |  | Fruit tea | 150 ml |
|  | Dinner | Potato | 150 g |
|  |  | Dairy-free margarine | 8 g |
|  |  | Broccoli | 40 g |
|  |  | Carrot | 40 g |
|  |  | Low-protein bread | 30 g |
|  |  | MSUD formula* | 20 g powder |
|  | Evening snack | Rice pudding (low protein milk substitute) | 120 g |
|  |  | MSUD formula* | 20 g powder |
| Day 3 | Breakfast | Low-protein toast | 40 g |
|  |  | Honey | 15 g |
|  |  | Dairy-free margarine | 5 g |
|  |  | Apple slices | 80 g |
|  |  | MSUD formula* | 20 g powder |
|  | Morning snack | Watermelon | 120 g |
|  |  | Fruit juice | 150 ml |
|  | Lunch | Low-protein noodles | 40 g |
|  |  | Carrot | 40 g |
|  |  | Potato | 50 g |
|  |  | Olive oil | 8 ml |
|  |  | Low-protein bread | 30 g |
|  |  | Banana pudding (plant milk) | 100 g |
|  | Afternoon Snack | Apple | 100 g |
|  |  | Low-protein crackers | 15 g |
|  | Dinner | Bell pepper | 100 g |
|  |  | Low-protein rice | 40 g |
|  |  | Tomato sauce | 60 g |
|  |  | Olive oil | 10 ml |
|  |  | Salad (cucumber + olive oil) (50 + 5 g) | 55 g |
|  |  | MSUD formula* | 20 g powder |
|  | Evening snack | Fruit jelly | 120 g |
|  |  | MSUD formula* | 20 g powder |

*Nutricia MSUD Anamix Junior ® (powder product – 20 g of powder is mixed with 150 ml of water).

**Appendix Table 4.** Three-day dietary plan generated by Gemini for a pediatric MSUD case

| Day | Meal | Food item | Amount/Grammage |
| --- | --- | --- | --- |
| Day 1 | Breakfast | Low-protein toast | 30 g |
|  |  | Margarine | 10 g |
|  |  | Strawberry jam | 7 g |
|  |  | Peaches | 50 g |
|  |  | Medical formula* | 30 g powder |
|  | Lunch | Low-protein pasta | 40 g (dry weight) |
|  |  | Olive oil | 15 ml |
|  |  | sautéed peppers/onions | 30 g |
|  |  | Apple juice | 150 ml |
|  | Afternoon snack | Low-protein crackers | 15 g |
|  |  | Honey | 10 g |
|  | Dinner | Vegetable soup (potato + carrot + celery + vegetable oil) (40 + 20 + 10 + 10 g) | 1 bowl (200 ml) |
|  |  | Medical formula* | 30 g powder |
| Day 2 | Breakfast | Low-protein cereal | 30 g |
|  |  | Coconut-based milk | 100 ml |
|  |  | Blueberries | 30 g |
|  | Lunch | White rice (dry) | 30 g |
|  |  | Butter | 10 g |
|  |  | Steamed cauliflower | 40 g |
|  |  | Medical Formula* | 30 g powder |
|  | Afternoon snack | Pear | 80 g |
|  | Dinner | Low-protein pizza (low-protein crust + tomato sauce + sautéed mushrooms + olives) (40 + 20 + 20 + 10 g) | 90 g |
|  |  | Medical Formula* | 30 g powder |
| Day 3 | Breakfast | Low-protein crepes (low-protein flour + egg substitute powder + olive oil + sugar + baking powder) (50 + 5 + 5 + 5 + 2 g) | 65 g |
|  |  | Maple syrup | 15 g |
|  |  | Medical formula* | 30 g powder |
|  | Lunch | Vegetable stir-fry: (zucchini + red cabbage + ginger + sesame oil) (50 + 20 + 10 + 15 g) | 95 g |
|  |  | Low-protein noodles | 30 g |
|  |  | Vegetable oil | 5 g |
|  | Afternoon Snack | Banana | 90 g |
|  | Dinner | Roasted sweet potato | 60 g |
|  |  | Margarine | 10 g |
|  |  | Cucumber salad (cucumber + vinegar + oil dressing) (40 + 5 + 5 g) | 50 g |
|  |  | Medical formula* | 30 g powder |

*Nutricia MSUD Anamix Junior ® (powder product – 30 g of powder is mixed with 225 ml of water).

**Appendix Table 5.** Three-day dietary plan generated by ChatGPT for a pediatric PPA case

| Day | Meal | Food item | Amount/Grammage |
| --- | --- | --- | --- |
| Day 1 | Breakfast | Low-protein bread | 40 g |
|  |  | Strawberry jam | 15 g |
|  |  | Unsalted butter | 5 g |
|  |  | Cucumber | 40 g |
|  |  | Olive oil | 5 ml |
|  |  | PPA formula* | 200 ml (53 g powder) |
|  | Morning snack | Apple | 120 g |
|  |  | Low-protein crackers | 15 g |
|  | Lunch | Low-protein rice | 150 g |
|  |  | Zucchini | 80 g |
|  |  | Carrot | 60 g |
|  |  | Olive oil | 10 ml |
|  |  | White bread | 15 g |
|  | Afternoon snack | Pear juice | 150 ml |
|  |  | Low-protein biscuit | 20 g |
|  | Dinner | Low-protein pasta | 160 g |
|  |  | Tomato sauce (homemade, low protein) | 80 g |
|  |  | Green salad (lettuce + cucumber) (40 + 40 g) | 80 g |
|  |  | Olive oil | 15 ml |
|  |  | Low-protein bread | 30 g |
|  |  | PPA formula* | 200 ml (53 g powder) |
| Day 2 | Breakfast | Low-protein pancakes (low-protein flour + sugar + vegetable oil) (40 + 10 + 5 g) | 55 g |
|  |  | Huney | 15 g |
|  |  | Mandarin | 100 g |
|  |  | Strawberries | 70 g |
|  |  | PPA formula* | 200 ml (53 g powder) |
|  | Morning snack | Banana | 80 g |
|  |  | Low-protein breadsticks | 20 ml |
|  | Lunch | Mashed potato (180 g + 10 g oil) | 190 g |
|  |  | Green beans | 80 g |
|  |  | Rice | 40 g |
|  |  | Olive oil | 5 ml |
|  | Afternoon snack | Peach juice 150 ml; | 150 ml |
|  |  | Low-protein cookie 20 g | 20 g |
|  | Dinner | Vegetable soup (potato + carrot + zucchini + cooked rice + olive oil) (70 + 50 + 60 + 20 + 10 g) | 300 ml |
|  |  | Low-protein bread | 30 g |
|  |  | Olive oil | 5 ml |
|  |  | PPA formula* | 200 ml (53 g powder) |
| Day 3 | Breakfast | Low-protein toast | 40 g |
|  |  | Apricot jam | 15 g |
|  |  | Butter | 5 g |
|  |  | Tomato | 60 g |
|  |  | Olive oil | 5 ml |
|  |  | PPA formula* | 200 ml (53 g powder) |
|  | Morning snack | Strawberries | 120 g |
|  |  | Low-protein crackers | 15 g |
|  | Lunch | Low-protein pasta | 150 g |
|  |  | Olive oil | 10 ml |
|  |  | Eggplant | 80 g |
|  |  | Tomato sauce | 70 g |
|  |  | White bread | 15 g |
|  | Afternoon Snack | Apple juice 150 | 150 ml |
|  |  | Low-protein biscuit | 20 g |
|  | Dinner | Vegetable rice pilaf (low-protein rice + carrot + zucchini + olive oil) (50 + 50 + 60 + 10 g) | 170 g |
|  |  | Lettuce salad | 60 g |
|  |  | Olive oil | 5 ml |
|  |  | PPA formula* | 200 ml (53 g powder) |

*Nutricia MMA/PA Anamix Next ® (powder product – a 200-ml solution is prepared using 53 grams of powder).

**Appendix Table 6.** Three-day dietary plan generated by Gemini for a pediatric PPA case

| Day | Meal | Food item | Amount/Grammage |
| --- | --- | --- | --- |
| Day 1 | Breakfast | Low-protein toast | 2 slices (60 g) |
|  |  | Butter | 10 g |
|  |  | Diluted fruit jam (strawberry) | 15 g |
|  |  | Medical formula* | 200 ml (53 g powder) |
|  | Morning snack | Apple slices | 80 g |
|  | Lunch | Vegetable soup (potato + carrots + zucchini) (30 + 20 + 40 g) | 150 ml |
|  |  | Olive oil | 20 ml |
|  |  | Low-protein pasta (dry) | 35 g |
|  |  | Tomato sauce | 40 g |
|  | Afternoon snack | Low-protein cookies | 20 g |
|  | Dinner | Roasted potato wedges (potato + olive oil) (115 g + 12 ml) | 100 g |
|  |  | Steamed broccoli florets | 30 g |
|  |  | Medical formula* | 200 ml (53 g powder) |
| Day 2 | Breakfast | Low-protein cereal | 30 g |
|  |  | Sliced banana | 40 g |
|  |  | Medical formula* | 200 ml (53 g powder) |
|  | Morning snack | Pear juice | 150 ml |
|  | Lunch | White rice (measured cooked) | 70 g |
|  |  | Sautéed bell peppers (20 g) and onions (20 g) in oil (20 ml) | 50 g |
|  |  | Medical Formula* | 100 ml (26.5 g powder) |
|  | Afternoon snack | Grapes | 60 g |
|  | Dinner | Low-protein burger with vegetables (lettuce + cucumber + tomato + ketchup + olive oil) (10 + 15 + 15 + 5 + 5 g) | 50 g |
|  |  | Mashed cauliflower (90 g + 10 g margarine) | 80 g |
|  |  | Medical formula* | 200 ml (53 g powder) |
| Day 3 | Breakfast | Low-protein pancakes (low-protein pancakes (low-protein flour + egg substitute powder + sunflower oil + medical formula) (25 + 3 + 5 + 5 g) | 38 g |
|  |  | Maple syrup | 15 g |
|  |  | Medical formula* | 200 ml (53 g powder) |
|  | Morning snack | Peach slices (canned or fresh) | 70 g |
|  | Lunch | Low-protein bread | 30 g |
|  |  | Avocado mash | 25 g |
|  |  | Olive oil | 15 ml |
|  |  | Vegetable vermicelli (vermicelli + carrots + zucchini + tomato juice) (15 + 15 + 35 + 10 g) | 75 g |
|  |  | Medical formula* | 150 ml (39.8 g powder) |
|  | Afternoon Snack | Fruit leather (mulberry) | 15 g |
|  | Dinner | Boiled sweet potato (dry 90 g) | 60 g |
|  |  | Stir-fried cabbage and mushrooms (cabbage + mushroom + olive oil) (45 + 15 + 12 g) | 60 g |
|  |  | Medical formula* | 200 ml (53 g powder) |

*Nutricia MMA/PA Anamix Next ® (powder product – a 200-ml solution is prepared using 53 grams of powder).
